# Effect of Saline Injection Mixing on Accuracy of Conductance Lumen Sizing of Peripheral Vessels

**DOI:** 10.1371/journal.pone.0074622

**Published:** 2013-09-13

**Authors:** Hyo Won Choi, Benjamin Jansen, David Birrer, Ghassan S. Kassab

**Affiliations:** 1 Department of Biomedical Engineering, Indiana University Purdue University, Indianapolis, Indiana, United States of America; 2 Department of Surgery, Indiana University Purdue University, Indianapolis, Indiana, United States of America; 3 Department of Cellular and Integrative Physiology, Indiana University Purdue University, Indianapolis, Indiana, United States of America; University of Arizona, United States of America

## Abstract

Transient displacement of blood in vessel lumen with saline injection is necessary in the conductance method for measurement of arterial cross-sectional area (CSA). The displacement of blood is dictated by the interactions between arterial flow hemodynamics and saline injection dynamics. The objective of the present study is to understand how the accuracy of conductance measurements is affected by the saline injection. Computational simulations were performed to assess the error in predictions of arterial CSA using conductance measurements over a range of peripheral artery diameters (i.e., 4, 7, and 10 mm) with an introducing catheter (6 Fr.) for various blood flow and saline injection rates. The simulation results were validated using the conductance measurements of the phantoms with known diameters (i.e., 7 and 10 mm). The results demonstrated that a minimum ratio of saline injection rate to blood flow rate of 3 is needed to fully displace the blood and result in accurate measurement of CSA for the peripheral artery sizes considered. Furthermore, the error was shown to be minimized as the detection electrodes are positioned between the distal to the mixing zone induced by saline injection and far downstream (4–8 cm from the injection catheter tip). The present study shows that even for the large peripheral arteries (7–10 mm) where mixing can occur, an appropriate injection rate and detection position can produce accurate measurement of lumen size.

## Introduction

The imaging-based technologies such as quantitative coronary angiography (QCA) and intravascular ultrasound (IVUS) have been adopted as the clinical standards [1] for measurement of vessel lumen. A physics-based, non-imaging method using a conductance catheter was introduced to address some of the inaccuracies of QCA and the limitations of inter- and intra-variability in interpretation of images and added time and cost for the determination of vessel lumen area using IVUS [2], [3]. Indeed, the electrical conductance method has been widely used for measurement of luminal area of aorta [4], [5] and medium size arteries [1]–[3], [6] as well as ventricular volume [7]–[11]. A two-injection approach was introduced by Kassab et al. [2], [3] to analytically determine a vessel lumen CSA by two bolus injections of saline solutions (e.g., normal and half normal) with known electrical conductivities. The saline flush to transiently displace the blood is fundamental for accurate measurement of vessel CSA. The saline of known conductivity must displace the majority of blood in the vessel lumen such that the electrical conductance of saline is measured during the injection duration (2–3 s).

The hemodynamic behavior of angiographic contrast injection into blood vessels has been extensively studied [12]–[18]. Similar analysis has not been carried out for saline flush of blood which is done very commonly in clinical practice. To ensure full displacement of blood and to understand the degree of mixing, one must understand the relation between injection rates and blood flows as well as the dimensions of the injection catheter relative to vessel size. Here, we performed computational fluid dynamics (CFD) simulations to investigate how the accuracy of CSA measurement is affected by blood-saline interactions over a variety of blood vessel sizes and blood/saline flow rates.

The present study provided a physical basis for the saline flush conductance method for determination of vessel lumen area. The numerical simulations were validated using *in vitro* conductance measurements in plastic tube cylindrical phantoms with known diameters. The findings of this study provide practical guidance on saline injection dynamics over a variety of blood vessels including large peripheral arteries where mixing is more prevalent.

## Methods

### Computational Domain and Flow Modeling

**Figure 1:**
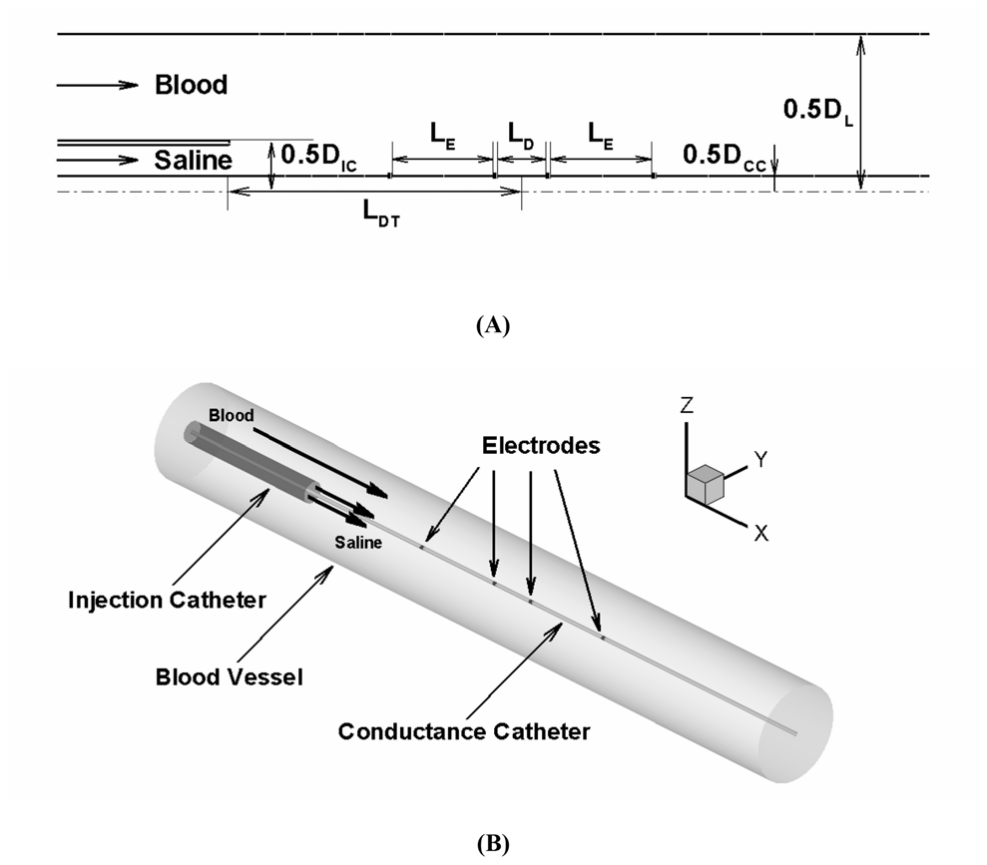
Schematic of the computational domain used for simulations. (**A**) 2-D axisymmetric view along the longitudinal direction and dimensions of vessel-injection catheter-conductance catheter configuration. (**B**) 3-D computational domain illustrating *in vitro* conductance catheter measurements. D_L_, D_IC_, and D_CC_ respectively denote the vessel lumen diameter, injection catheter diameter, and conductance catheter diameter. In the present simulations, the injection and conductance catheter diameter were assumed to be 2 mm (corresponding to 6 Fr. guide-catheter) and 0.9 mm, respectively (corresponding to 0.035” guide-wire). L_E_, L_D_, and L_DT_ respectively denote the excitation electrode distance (set as 4 mm), detection electrode distance (set as 1 mm), and distance of detection electrode from the injection catheter tip. Note that the excitation electrode spacing was set as 20 mm for the larger vessel sizes considered (i.e., 7 and 10 mm).

All simulations were performed for the 3-D computational domains depicted in Fig. 1. The saline injection rate and arterial blood flow rate were assumed such that the ratio of saline injection rate to blood flow rate was varied from 1 to 5 over a wide range of vessel diameters (4, 7, and 10 mm). The 10 ml of saline solution was assumed to be injected into an artery through a commonly used catheter dimension (i.e., 2 mm lumen diameter corresponding to 6 Fr.) for 2–10 s (corresponding to 1–5 ml/s of saline injection rate) in order to determine the effect of saline injection and mixing dynamics on conductance catheter performance. The flow conditions and simulation parameters used in the present study are listed in Table 1.

**Table 1:** Flow Conditions and Physical Parameters used in Simulations.

**Blood Vessel Diameter [mm]**	4	7	10	**Refs.**
**Injection Catheter Size [Fr.]**	6			
**Blood Density [kg/m^3^]**	1050			27
**Blood Viscosity [cP]**	3.5			28, 29
**Saline Density [kg/m^3^]**	1000			assumed to be similar to water
**Saline Viscosity [cP]**	0.9			
**Blood Electrical Conductivity [S/m]**	0.69			4
**Saline Electrical Conductivity [S/m]**	0.701 (0.45% Saline) and 1.316 (0.9% Saline)			Measured

### Numerical Modeling for Flow and Mass Transport

The injected saline solution interacts with the blood flow in vessel lumen in a complex manner depending on the blood and saline flow dynamics. Complex flow patterns and turbulent flow regime may be involved. Flow and mass transport fields were obtained by solving the Navier-Stokes equation with turbulence models and convection-diffusion equation utilizing ANSYS FLUENT 12.1 (ANSYS Inc., Lebanon, NH) as follows:
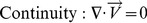
(1)

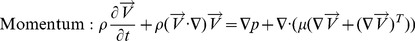
(2)

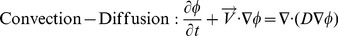
(3)where 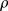, 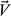, 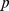, 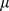, 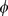 and 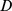 denote density, velocity, pressure, dynamic viscosity, saline mass fraction, and diffusivity, respectively. The density and viscosity were assumed to be linearly weighted between blood and saline depending on the saline mass fraction that changes with space and time. The k-ε model provided by FLUENT (the realizable k-ε model proposed by Shih et al., ANSYS FLUENT 12.0 Theory Guide) was adopted to describe turbulent flow behavior.

Non-slip boundary conditions were applied on the vessel and catheter walls. A uniform velocity profile was imposed, respectively, at the lumen and injection catheter inlet. A constant saline mass fraction was specified as 0 and 1 at lumen and injection catheter inlet, respectively. At the outlet, the outflow boundary condition (i.e., fully developed condition) was applied for both flow and mass transport field. The simulations were verified to be mesh-independent.

### Numerical Modeling for Electric Field

In order to obtain the electric field in vessel lumen, the Poisson’s equation was solved subject to the boundary condition of zero electric potential gradients at all walls and driving current at excitation electrodes as follows:
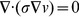
(4)where 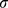 and  denote respectively, the electrical conductivity and the electric potential. As described in the previous studies [2], [3], CSA is analytically determined using two injections of different saline solutions with the following equation:
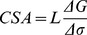
(5)where 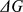, 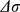 and 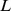 respectively denote the conductance change for two saline injections, the electrical conductivity difference between two saline solutions and the distance between detection electrodes. The percent error in diameter, ε, is defined as 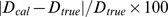 where 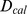 and 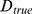 respectively denote calculated diameter from conductance CSA and true diameter [3].

### Equivalent Conductivity in Lumen

The electrical conductivity of vessel lumen is dependent on how the injected saline solution displaces blood, which is inevitably determined by the mixing dynamics between saline and blood. The electrical conductivity at a given position of vessel lumen was assumed to be a function of saline mass fraction (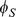), which varies with space (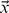) and time (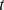) depending on the flow field and mixing dynamics, as follows:
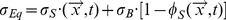
(6)where the subscripts *Eq*, *S*, and *B* denote ‘equivalent’, ‘saline’, and ‘blood’, respectively. Since the electrical conductivity of saline solution is linearly proportional to salinity, the present modeling states that the equivalent conductivity is a linearly weighted average of saline and blood.

As described in Eq. (5), 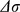
reflects the electrical conductivity difference (i.e., 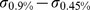). Once the saline solutions are injected into vessel lumen, they are dynamically mixed with blood such that the resulting electrical conductivities of lumen governed by Eq. (6) may deviate from their initial states (i.e., 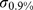 and 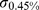) depending on the extent of mixing. Although normal (0.9%) and half normal (0.45%) saline solutions have identical fluid properties in terms of mixing with blood, the difference in electrical conductivity 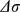 used for determination of CSA can give rise to certain degree of error.

### Experimental Validation

The computer simulations were validated with *in vitro* conductance measurements of the plastic phantom tubes with known diameter of 7 and 10 mm (n = 3 for each diameter) as illustrated in Fig. 1B. A 0.45% saline solution was perfused into the phantom tube as a baseline circulating medium and 0.45% and 0.9% saline solutions were injected through a 6 Fr. sheath inserted into the phantom tube. The conductance was then measured at the various axial positions of the tube (i.e., 2–12 cm distance of detection electrodes position relative to the sheath tip) over a wide range of flow rate ratios (i.e., 2, 2.5, 3.3, and 5).

## Results

### Saline Injection Flow Patterns

Displacement of blood by injected saline solution is an essential feature of the determination of artery diameter by conductance measurements because the blood displacement is directly associated with the accuracy of CSA calculation through the conductivity difference 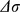 as in Eq. (5). Therefore, it is important to investigate how effectively blood is displaced by the injected saline solution, which is determined by the relative mixing dynamics between blood and saline flows.

**Figure 2:**
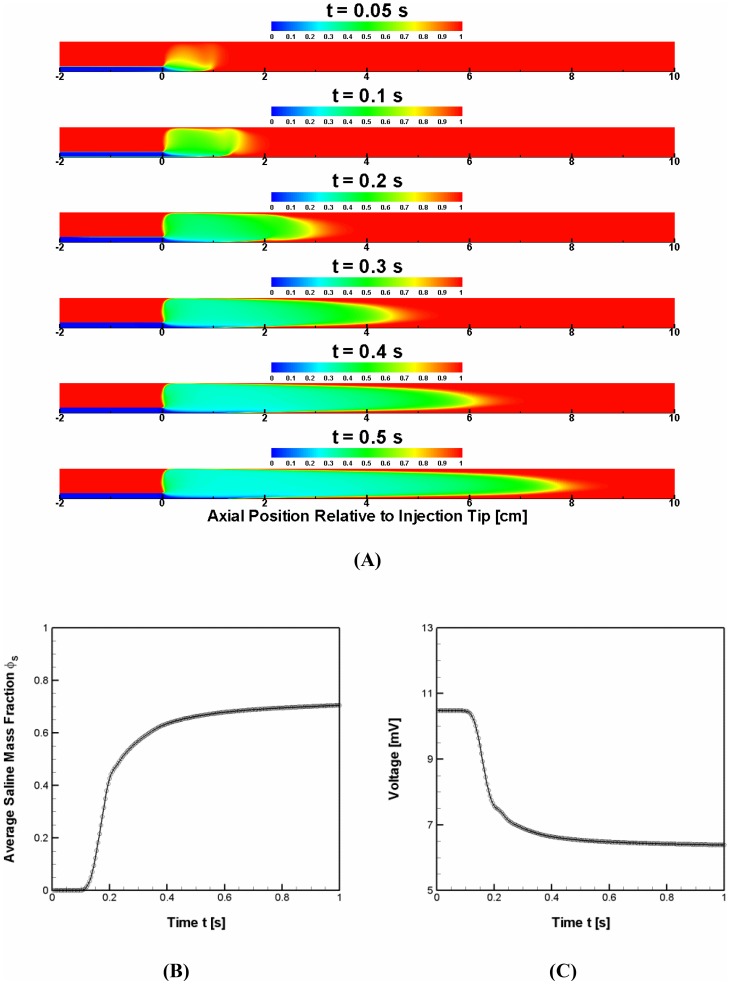
Temporal evolution of (A) saline fraction field, (B) average saline fraction over the cross-section at detection electrodes center, and (C) corresponding voltage detection for the 7 mm diameter vessel (i.e., D_L_ = 7 mm) at flow rate ratio of 3 (i.e., 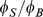 = 3). Red and blue colors denote pure blood and saline, respectively. Saline was assumed to be injected at t = 0 s. The voltage detection position (i.e., center of detection electrodes) is 20 mm from the injection catheter tip. Four black dashed lines in (A) denote the positions of excitation (outer pair) and detection (inner pair) electrodes.

Figure 2A depicts the temporal evolution of saline fraction field after injection. Figures 2B and 2C demonstrate the temporal profile of saline fraction averaged over the cross section of lumen at detection electrodes center (2 cm from injection catheter tip) and the voltage detected, respectively. The results show that the voltage begins to deflect as the injected saline is transported to and sensed by the detection electrodes (> t = 0.1 s). Then, the voltage deflection reaches a plateau after the saline displaces the blood over the whole electrodes region in lumen (> t = 0.3 s). The majority of major changes in saline fraction and voltage deflection occur between the two time points which vary depending on the detection position and blood/saline flow dynamics.

**Figure 3:**
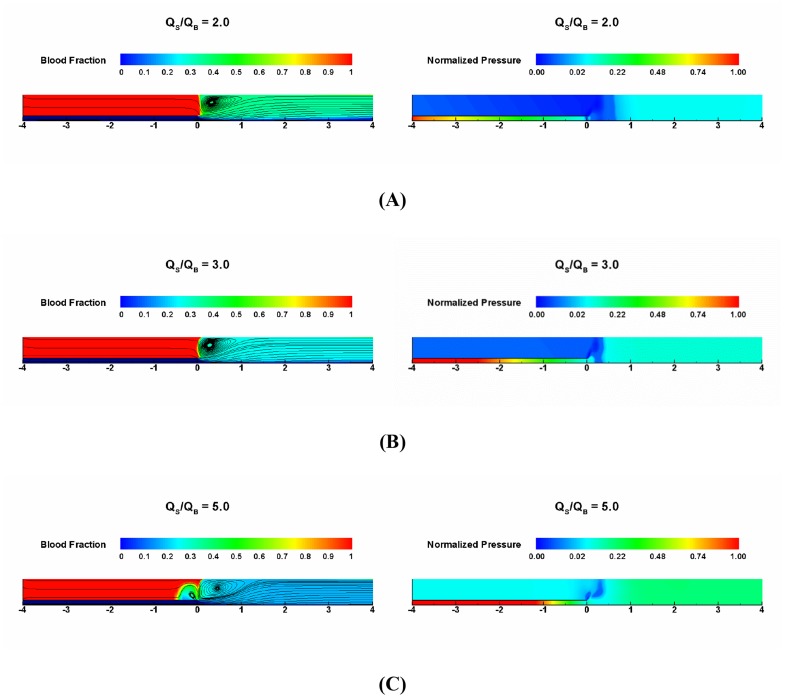
Streamlines and blood fraction contours (left panels) and pressure contours (right panels) at the flow rate ratio of (A) 2, (B) 3, and (C) 5. The vessel lumen diameter D_L_ is 7 mm.

The left panels of Fig. 3 demonstrate the instantaneous streamlines and corresponding blood fraction 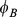 (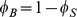 where 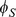 is saline fraction) contours over a wide range of saline to blood flow rate ratios (i.e., 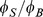 = 2, 3, and 5). It is apparent that the injected saline solutions present a pressure “wall” so that the blood flowing from upstream is blocked and the injected saline solutions transiently displace the blood. The magnitude of the injection rate relative to blood flow determines the degree of pressure wall such that different extent of blood flow recirculation may occur. The results indicate that the mixing flow pattern including recirculating bubble size becomes intensified as the flow ratio increases (see left panels of Fig. 3). This is also consistent with the right panels of Fig. 3 which demonstrate that the pressure barrier to blood flow induced by saline injection increases as the flow ratio increases.

### Effect of Injection Dynamics on Conductance Catheter Performance

**Figure 4:**
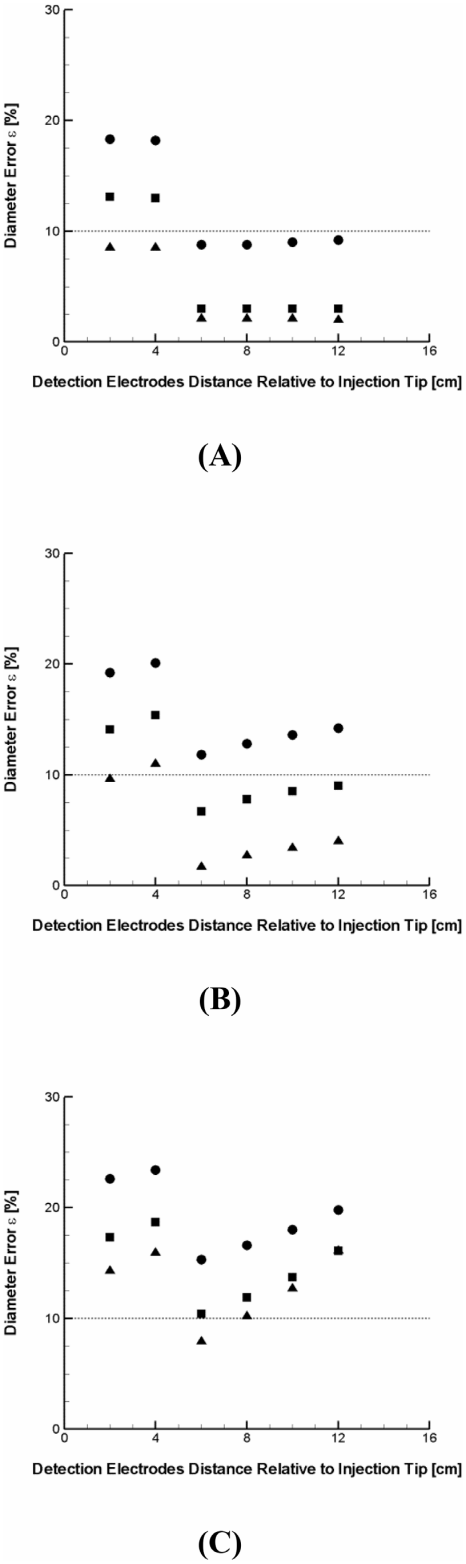
Percent error in predicted diameter for three different ratios of saline injection rate to blood flow rate (i.e., 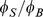 = 2, 3, and 5) along the various axial positions of conductance catheter relative to injection catheter for a variety of lumen diameters (A) D_L_ = 4 mm, (B) D_L_ = 7 mm, and (C) D_L_ = 10 mm with a commonly used injection catheter size (i.e., D_IC_ = 6 Fr.). Circle, rectangle, and triangle symbols denote the ratio of 2, 3, and 5, respectively. The detection electrodes distance relative to injection tip indicates the distance of detection electrodes center from the distal end of injection catheter.

The mixing dynamics of injected saline solution with blood in conductance measurement affects the electrical conductivity and associated electric field of vessel lumen. Figure 4 depicts the percent error in diameter predicted by conductance measurements for a variety of lumen diameters (i.e., 4, 7, and 10 mm) and for a commonly used injection catheter size (i.e., 2 mm diameter corresponding to a 6Fr. lumen) with three different ratios of saline injection rates to blood flow rate (i.e., 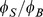 = 2, 3, and 5) along the various axial positions of conductance catheter.

The results demonstrated that the error in predicted diameter generally decreases as the ratio increases regardless of the vessel size and detection position considered. The improvement in accuracy with the flow ratio, however, becomes more prominent as the vessel size decreases (Fig. 4C Fig. 4B Fig. 4A). The results also demonstrated that the error is generally higher when the conductance catheter electrodes are located in the vicinity of injection catheter tip (<4 cm) where flow is highly disturbed and complex mixing occurs. Such variability in the error with the catheter position seems to be common regardless of the vessel size and flow rate ratio considered. The results show that the error can be reduced to 9%, however, at the detection electrodes positions relative to injection catheter tip>4 cm (i.e., downstream of mixing zone) for the 4 mm lumen diameter once the flow ratio exceeds 2. The error was shown to decrease to 7% at the flow rate ratio of 3 for the 7 mm lumen diameter. For the 10 mm lumen diameter, the level of error (<12%) was shown to be attainable at the flow rate ratio >3 only within a limited range of detection positions (i.e., 4–8 cm). Furthermore, the error was 9% even within the region <4 cm for the ratio of 5 when the vessel is in a medium size artery range (i.e., 4 mm) while the higher flow rate ratios was not shown to reduce the measurement error to the desired level (i.e., <10%) in the vicinity of disturbed flow region as the vessel size becomes larger (i.e., 7 and 10 mm).

**Figure 5:**
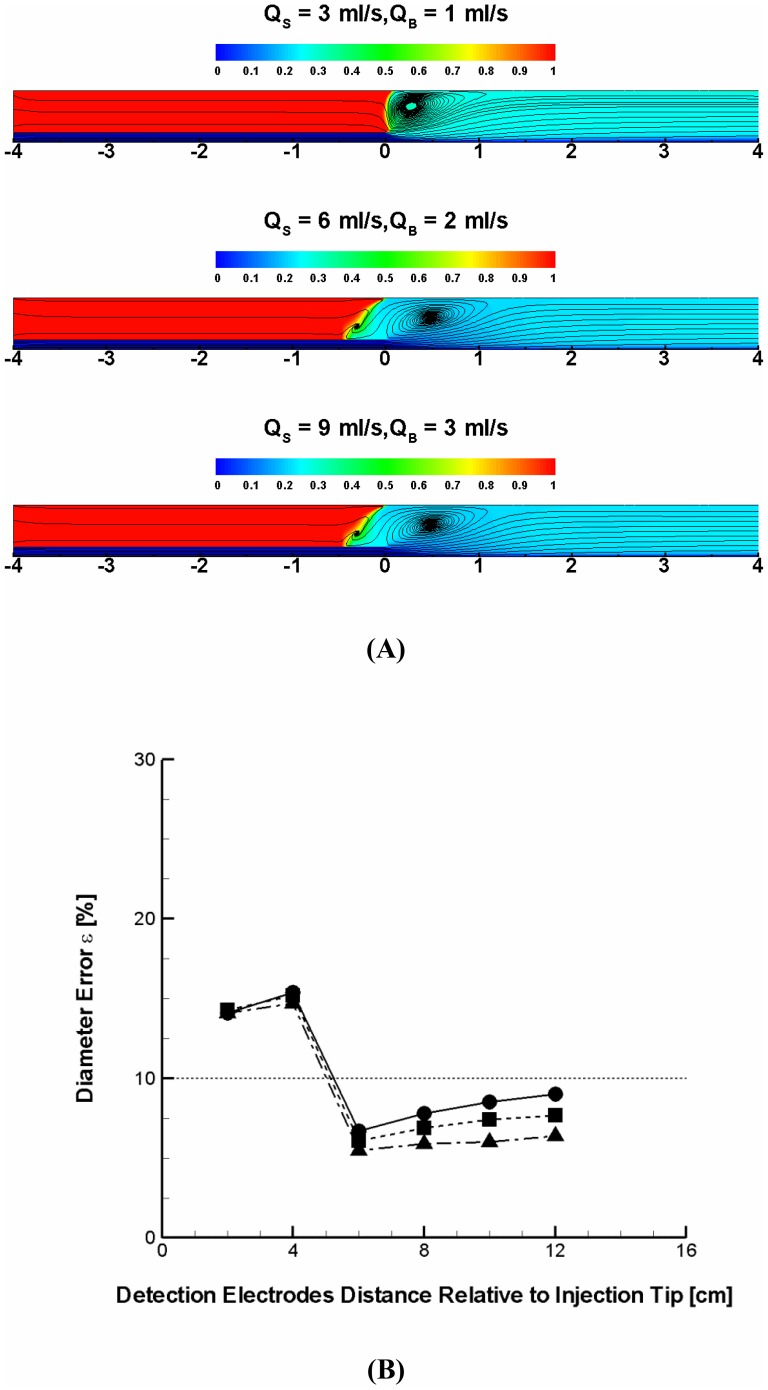
Effect of combinations of saline injection and blood flow rate on conductance measurement at a given flow rate ratio. (**A**) Streamlines and blood fraction contours in the 7 mm diameter vessel at the flow rate ratio of 3 for three different combinations of saline injection and blood flow rates (i.e., Q_S_ = 3 ml/s and Q_B_ = 1 ml/s, Q_S_ = 6 ml/s and Q_B_ = 2 ml/s, and Q_S_ = 9 ml/s and Q_B_ = 3 ml/s). (**B**) Percent error in diameter corresponding to the three different flow rate conditions. Circle, rectangle, and triangle symbols denote the flow condition of Q_S_ = 3 ml/s and Q_B_ = 1 ml/s, Q_S_ = 6 ml/s and Q_B_ = 2 ml/s, and Q_S_ = 9 ml/s and Q_B_ = 3 ml/s, respectively.

Arterial blood flow rate has been extensively measured using a variety of methods. The volumetric blood flow rate varies in arteries [19]–[22] and depends on the disease status of arteries with stenosis [19], [20]. Based on the previously measured values of volume flow rates in arteries, it was determined how the different combinations of flow rates with an identical flow rate ratio can affect the conductance measurements. Figure 5A depicts the streamlines and saline fraction contours for three different flow rate conditions resulting in a same flow rate ratio (i.e., 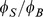 = (3 ml/s)/(1 ml/s), (6 ml/s)/(2 ml/s), and (9 ml/s)/(3 ml/s)). The results demonstrated that the degree of flow disturbance in mixing zone increases and reaches a plateau as both blood and saline flow rates increase. The changes in saline fraction distribution distal to the injection catheter tip, however, seem to be minimal. In fact, the deviation in error was shown to be virtually negligible despite the different flow rate conditions as long as the flow rate ratio remains the same as apparent in Fig. 5B. The results suggest that the ratio of saline injection to blood flow rate is the critical determinant of how effectively the injected saline blocks and displaces blood rather than the magnitude of saline injection or blood flow rate for the artery lumen diameters considered.

### In Vitro Validation

**Figure 6:**
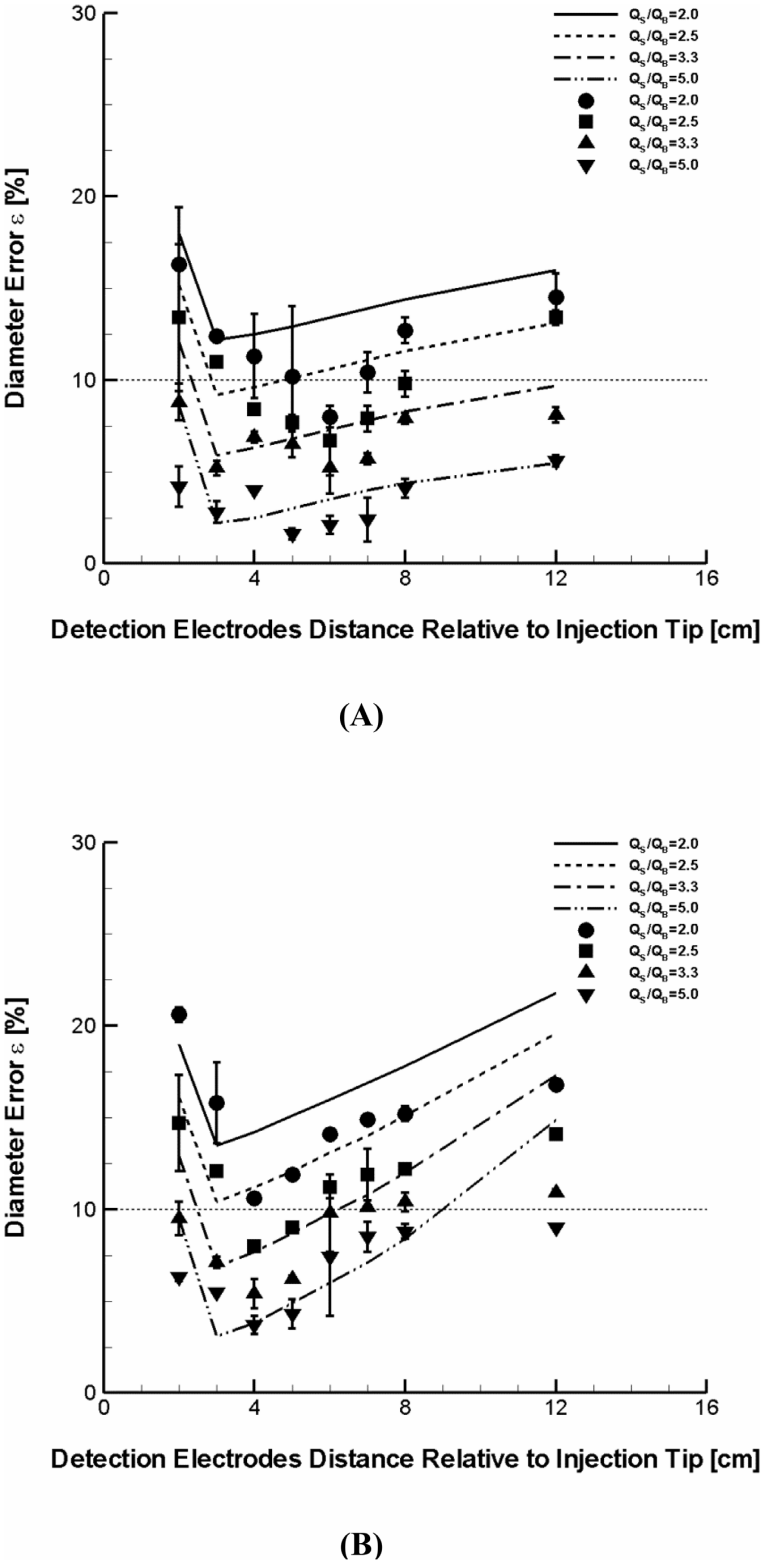
Comparison of the percent error in diameter calculated by simulations and conductance measurements of phantoms at four different ratios of injection rate to baseline flow rate (i.e., 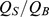 = 2, 2.5, 3.3, and 5) for two different sizes (A) D_L_ = 7 mm and (B) D_L_ = 10 mm. Lines and symbols represent simulations and measurements, respectively. Circle and solid, rectangle and dashed, triangle and dashed-dot, and gradient and dashed dot-dot denote the flow rate ratio of 2, 2.5, 3.3, and 5, respectively. Half normal saline was used as a baseline flow medium. Each conductance measurement was made in three (n = 3) phantoms for each diameter. The largest error was found to be 4 and 3.2% for 7 and 10 mm diameter phantom, respectively.

The computer simulations were experimentally validated with *in vitro* conductance measurements in 7 and 10 mm diameter phantom tubes. As shown in Fig. 6, the results suggest that the computer simulations are generally in good agreement with the experimental measurements although the level of error is somewhat overestimated by the simulations. Specifically, the maximum difference in diameter error between simulations and experimental measurements over all flow rate ratios and detection positions considered were shown to be 5.4 and 6.4%, respectively for 7 and 10 mm diameter phantom tubes. Furthermore, the mean %error, averaged over all the positions measured as shown in Fig. 6A (D_L_ = 7 mm), were 14.2±1.8% in simulation predictions and 12.0±2.4% by conductance measurements for the flow rate ratio of 2. For the flow rate ratios of 2.5, 3.3 and 5, the mean %errors were 11.3±1.9% vs. 9.8±2.4%, 8.0±1.9% vs. 6.8±1.3%, and 4.2±1.9% vs. 3.4±1.3%, respectively. The corresponding mean %errors for the 10 mm vessel (Fig. 6B) were 16.8±2.6% vs. 15.0±2.9%, 14.0±2.8% vs. 11.7±2.1%, 10.8±3.1% vs. 8.7±2.0%, and 7.2±3.6% vs. 6.7±1.9%.

The present simulations are largely consistent with the experiments that the measurement accuracy is improved for both phantom sizes as the ratio of saline injection rate to baseline flow rate increases. Specifically, similar to the computational simulations, the results demonstrate that once the flow rate ratio exceeds a certain threshold (e.g., >3), the error can be reduced down to an acceptable level (i.e., <10%) even for the largest artery size considered (i.e., D_L_ = 10 mm). The phantom conductance measurements for each diameter (n = 3) showed that *in vitro* measurement is highly reproducible such that variability in error is <1% over the majority of measurements albeit the maximum variability was no greater than 4 and 3.2%, respectively for 7 and 10 mm diameter phantom tubes.

## Discussion

The saline injection technique is used to displace blood with a known conductivity for the determination of artery lumen diameter using the conductance catheter [1]–[3], [6]. The saline injection inherently involves the mixing of injected saline solution with blood. Depending on the blood and saline flow conditions, the mixing dynamics between saline and blood can result in adequate displacement of blood which can lead to measurement error. Here, we assessed how the accuracy of conductance measurement is affected by the saline-blood mixing for a range of blood vessel diameters (i.e., D_L_ = 4, 7, and 10 mm) and the commonly used injection catheter size (i.e., D_IC_ = 2 mm corresponding to 6 Fr.) subject to a wide range of saline injections and blood flow rates.

The present findings on saline injection flow patterns were similar to those of contrast injection [15]. The blood flow can be blocked by the injected solution depending on the ratio of saline or contrast injection rate to blood flow rate (Fig. 3). This was attributable to the different degree of pressure barrier provided by saline injection (right panels of Fig. 3). Such notion is consistent with previous studies which showed that the magnitude of the generated back pressure and hence the degree of blood displacement depends on the ratio of contrast injection to blood flow [23], [24].

The results demonstrate that the CSA accuracy can be affected by the relative flow conditions between blood and saline. Specifically, the error was shown to significantly decrease as the flow rate ratio of saline to blood increases (Fig. 4). The results suggest that the extent of blood displacement and hence conductance catheter performance become nearly independent of flow rate ratio beyond a certain threshold in that the error is reduced to <10% (i.e., 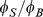 >3, Fig. 4). The critical threshold, however, seems to slightly increase as the lumen diameter increases (i.e., 2 for 4 mm, 2.5 for 7 mm, and 3 for 10 mm, Figs. 4 and 6). In fact, the ratio of 2 for 4 mm lumen diameter is in good agreement with the previous study which showed that the ratio of 2 was required to completely displace the circulating flow by contrast injection for a 6 mm diameter phantom [23]. It would be an interesting future study to evaluate the non-linear pattern in error prediction and critical threshold as the artery dimensions include the aorta.

Also, the results indicate that the error is generally higher when the conductance catheter electrodes are located proximal to the injection catheter tip (i.e., in the vicinity of mixing zone) than when they are placed distal to the tip. The results demonstrated that the higher flow rate ratios can effectively reduce the error (i.e., 9%), however, even proximal to the injection catheter tip for the smaller vessel size (i.e., 4 mm, Fig. 4A). In contrast, it was shown that the higher flow rate ratios cannot substantially improve the accuray within the mixing zone for the larger vessel sizes (i.e., 7 and 10 mm, Figs. 4B and 4C). Nevertherless, the error was shown to be measurable (<12%) for the optimal detection positions (4–8 cm) at the flow rate ratio >3 regardless of the vessel size considered.

The numerical simulations assessed how the relative mixing dyanmics and related conductance catheter performance are affected by a range of arterial flow rates published in the literature (i.e., 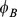 = 1–3 ml/s, Fig. 5B; refs. 19–22). The arterial flow rate is affected by the flow resistance which depends on degree of lumen stenosis. Although the impact of flow resistance in stenotic arteries on the arterial flow rate has not been taken into account as in previous studies [25], [26], the range of arterial flow rates adopted is conservative in that the flow rate decreases in stenotic arteries and this increases the ratio of saline to blood flow rate. The results demonstrated that the accuracy of vessel diameter predictions is largely insensitive to the variations in arterial volume flow rates as long as the ratio of saline injection rate to blood flow rate remains sufficiently high. The results suggest that the ratio rather than individual flow rates is the major determinant of the displacement of blood and hence the conductance accuracy. The present results suggest that the flow rate ratio >3 can be sufficient for the reliable blood displacement over a wide range of artery sizes (4–7 mm).

The effect of blood flow pulsatility on condutance measurement can be minimized as long as the saline injection rate is sufficiently higher than blood flow (e.g., the flow rate ratio >3, data not shown). This is because the pressure barrier generated by higher saline injection rates diminshes the flow pulsatility. Moreover, two bolus injections (i.e., normal and half normal) can offset the impact of flow pulsatility on conductance measurment accuracy since the two injections are hemodynamically similar. The effect of a smaller introducing catheter (i.e., 5 Fr.) on conductance catheter performance was also assessed. The results suggest that the effect of guide-catheter size (i.e., 5 and 6 Fr.) is minor since the difference in diameter error prediction was shown to be negligible (<2%, data not shown) for an optimal flow rate ratio (i.e., >3) and an adequate detection position (i.e, 4–8 cm).

The vessel compliance was not taken into account because the vessel lumen CSA is generally performed in diseased vessels that tend to be calcified and rigid. Furthermore, the influence of vessel compliance on CSA measurement accuracy is minimized by the two injection approach; i.e., the vessel (if compliant) would be distended and maintained to the same degree through the two injections. It may be necessary to examine the role of the vessel wall compliance on conductance measurement, however, for large elastic vessels including the aorta in future studies.

Turbulent flow is random three-dimensional phenomenon and very challenging to simulate. In spite of the variations in error of diameter predictions for the turbulence models considered at the low flow rate ratio (data not shown), the simulation results were shown to be consistent with the *in vitro* validation. The *in vitro* conductance measurements of phantoms confirmed that the higher flow rate ratios (>3) can significantly improve the accuracy in sizing (i.e., error <10%) even for the larger artery sizes (i.e., 7 and 10 mm) when the detection electrodes are placed appropriately (i.e., 4 cm<distance from injection catheter tip<8 cm, Fig. 6).

The current findings are consistent with previous studies [1]–[3], [6] that demonstrate the two injection method is an accurate method for determination of the lumen diameter of medium size arteries (2–4 mm coronary dimensions) since the majority of blood can be effectively displaced by the injected saline solution. Since the mixing issue is minimal for coronary blood vessel caliber (i.e., the injection rates of 10 ml saline in 2–3 s is significantly higher than coronary flow), we focused on larger (peripheral) vessels with higher blood flows. The present finding suggests that the higher ratio of injection rate to blood flow rate (i.e., 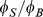 >3) is adequate to provide an accurate conductance CSA measurements even for the larger peripheral vessels.
